# Growth, yield and oil quality of adult pedestrian olive orchards grown at four different planting systems

**DOI:** 10.3389/fpls.2024.1416548

**Published:** 2024-07-19

**Authors:** Roberto Massenti, Antonino Ioppolo, Alessandro Carella, Valeria Imperiale, Riccardo Lo Bianco, Maurizio Servili, Roberto Selvaggini, Tiziano Caruso

**Affiliations:** ^1^ Department of Agricultural, Food and Forest Sciences, University of Palermo, Palermo, Italy; ^2^ Department of Agricultural, Food and Environmental Sciences, University of Perugia, Perugia, Italy

**Keywords:** canopy volume, fatty acids, Olea europaea, phenols, training form, yield efficiency

## Abstract

This study evaluated growth, yield and olive oil quality of mature pedestrian olive orchards. Trees of three Sicilian cultivars Calatina, Nocellara del Belice and Abunara were planted at four combinations of planting densities and training forms. Trees at 2 × 5 m were trained to central leader (CLx2), those at 3 × 5 m to free palmette (FPx3), those at 4 x 5 to globe vase (GVx4), and those at 5 x 5 to poly-conic vase (PVx5). ‘Calatina’ had the smallest trees in terms of trunk size in all growing systems, while canopy size of trees at higher densities was similar for all three cultivars. ‘Calatina’ was also the most growth efficient (m3 of canopy per cm2 of TCSA) and produced the least amount of pruning wood in the hedgerow systems (CLx2 and FPx3). Fruit yield per tree tended to be higher in more vigorous cultivars (Abunara and Nocellara) grown to 3D systems (GVx4 and PVx5), while ‘Calatina’ was the most yield efficient (kg of fruit per cm^2^ of TCSA) especially in the hedgerow growing systems. Fruit and oil yield per ha and average production value tended to be highest in CLx2 trees and lowest in GVx4 trees, with ‘Calatina’ showing the sharpest changes and ‘Nocellara’ the smallest changes among growing systems. According to the Jaen index, CLx2 tended to induce earlier fruit maturation, followed by PVx5, GVx4, and FPx3. The growing system did not affect oil fatty acid composition, while ‘Calatina’ had the highest amount of mono-unsaturated fatty acids and the lowest amount of saturated fatty acids. ‘Abunara’ oils exhibited the highest amount of total phenols in CLx2, while ‘Calatina’ and ‘Nocellara’ oils exhibited the highest amount in FPx3 and PVx5. Both, trans-2-hexenal (“cut grass” sensory note) and hexenyl acetate (“floral” sensory note) tended to be lowest in oils from trees grown at CLx2 and highest in those from trees grown at GVx4, showing a somewhat inverse relationship with fruit ripening degree. The outcome of the present study on mature pedestrian orchards shows that proper combinations of cultivars, planting densities, and training forms (canopy shape) may result in efficient intensive systems for growing olive in areas where super-high density systems cannot be profitable due to agronomic and environmental limitations (water shortage, steep sloping sites, small farm size, etc.). Pedestrian growing systems can also be used to exploit olive biodiversity by allowing the use of available local genotypes. For this reason, they may represent an effective and sustainable solution against unexpected climate changes and associated emerging diseases.

## 1 Introduction

In olive growing, harvesting and pruning require the highest economic investment from olive growers, and the only way to obtain a profitable return is to mechanize these two cultivation techniques (Navarro et al., 2017; Mairech et al., 2020). For this reason, traditional non-irrigated, low-density and manual-worked olive groves are being replaced by high-density and irrigated orchards designed for mechanization. This transformation has resulted in adaptations regarding cultivar selection, training forms, planting layouts, and cultivation techniques (Lo Bianco et al., 2021; Massenti et al., 2022a).

In Spain, the use of super-high-density (SHD) olive groves, with planting densities higher than 1300 trees/ha (Lo Bianco et al., 2021), has been very successful both agronomically (Taguas et al., 2021) and economically (Fernández et al., 2020). The benefits of this design include easier disease and pest control, improved irrigation and fertigation, and most notably, the cost-effective and efficient mechanized harvesting and pruning techniques (Tous et al., 2010). However, of the over six hundred olive cultivars certified and listed in the World Bank of Olive Germplasm (Trujillo et al., 2014; Belaj et al., 2020), only 4-6 are suitable for SHD olive orchards. Among those, only ‘Arbequina’, ‘Arbosana’, ‘Koroneiki’, ‘Sikitika’ and more recently ‘Oliana’ and ‘Lecciana’ (Camposeo et al., 2021, 2022) characterized by early bearing, high and constant productivity, and medium-low vegetative vigor, are the most widely grown and best performing cultivars in SHD planting systems (Tous et al., 2003; Centeno et al., 2019; Rodrigues et al., 2021). This could cause an olive biodiversity depletion and the loss of local and neglected cultivars that produce unique oils and are better adapted to specific soil and climate conditions or even more resistant to environmental stress due to climate change and the emergence of new disease and pest attacks.

Different studies conducted in central and southern Italy (Camposeo and Godini, 2010; Farinelli and Tombesi, 2015; Marino et al., 2017) have investigated the growth and production of some Italian varieties in comparison with ‘Arbequina’ in SHD systems. Except for ‘Urano’ and ‘Calatina’, due to their low vigor and high productivity, and ‘Abunara’ and ‘Cerasuola’, for the excellent quality of the oils produced, no other cultivar has shown to be suitable for SHD olive orchards. Duarte et al., 2008 and Lo Bianco et al., 2021 showed that in some areas of the Mediterranean, especially south Italy and Portugal, the use of SHD olive orchards is limited due to environmental conditions, steep sloping sites and water availability. Previous studies have shown that pedestrian olive orchards using selected local cultivars and different planting density and training forms for different types of mechanization may represent promising alternatives to SHD systems in those areas where SHD systems cannot be established (Marino et al., 2019; Lo Bianco et al., 2021; Massenti et al., 2022b).

Adopting the pedestrian growing systems would have indeed several advantages over SHD systems, at least in the areas mentioned above. For example, olive trees have a monumental, historical and environmental relevance (Anestiadou et al., 2017) and the implementation of pedestrian olive orchards would lead to an improvement in the biodiversity and sustainability of the olive-growing scenario, using local cultivars that are less sensitive to abiotic and biotic stressors (Saponari et al., 2019). In addition, the planting density and training forms adopted in pedestrian systems in accordance with the cultivar growth habit and vigor would allow appropriate and efficient mechanization of some onerous management techniques such as pruning and harvesting (Lo Bianco et al., 2021). Furthermore, it is generally accepted that for the same canopy volume, the free-palmette training system intercepts more sunlight due to its better surface area-to-volume ratio compared to the central-leader system typically used in SHD systems (Lo Bianco et al., 2021). This favors photosynthesis and increases production in terms of quality and quantity (Díez et al., 2016). Although a linear increase in yield in relation to planting density has been observed in several studies (Diez et al., 2015), in arid areas characterized by low water availability, increasing planting density may lead to high root competition for water and nutrients and affect plant productivity, ultimately reducing the overall quantity and value of olive oil production (Trentacoste et al., 2015). This aspect poses serious doubts on the long-term sustainability of SHD systems, especially in those areas characterized by limiting environmental factors.

In Sicily, the use of some local (major and minor) cultivars like Calatina, Nocellara del Belice and Abunara in pedestrian growing systems has been studied during the early stages of orchard life with interesting results in terms of fruit yield and olive oil quality (Marino et al., 2019; Lo Bianco et al., 2021; Carella et al., 2022; Massenti et al., 2022a). Different studies have shown the unique composition of Sicilian olive oils above all in terms of oleic, linoleic, palmitic acids, and their ratio, monounsaturated fatty acids, tocopherols, and total sterol content (Tripoli et al., 2009; Marino et al., 2019; Sicari et al., 2021; Massenti et al., 2022b). High contents of bioactive compounds in the olive oil have been associated to lower disease incidence or disease prevention in humans, adding further value to the unique composition of high-quality Sicilian olive oils (Pavone et al., 2010).

The aim of this study was to evaluate growth and yield performance, as well as olive oil quality, of pedestrian orchards at the beginning of their mature stage using different Sicilian cultivars, planting densities, and training forms. In particular, the trial is a combination of three Sicilian olive cultivars, four planting densities and four training forms, i.e. four growing systems. The study is a follow up of previous research on the same trees at the young stage (Massenti et al., 2022a), and will be a useful confirmation of results obtained from trees at the adult stage and in full production.

## 2 Materials and methods

### 2.1 Plant material and experimental conditions

The trial was conducted in a 2.79-ha experimental field located in southwest Sicily (37°31’ N, 13°03’ E, about 120 m a.s.l.) from 2021 to 2023. Seven-year-old olive trees of the Sicilian cultivars Abunara, Calatina (minor cultivars), and Nocellara del Belice (named Nocellara for brevity in the rest of the article; widespread in western Sicily and grown for producing both table olives and olive oil) were used for the trial. Trees were planted in north–south-oriented rows at four planting densities: 2 × 5 m (1000 trees/ha), 3 × 5 m (666 trees/ha), 4 × 5 m (500 trees/ha), and 5 × 5 m (400 trees/ha). Trees at 2 × 5 m were trained to central leader (CLx2), while those at 3 × 5 m were trained to free palmette (FPx3). Both these tree shapes formed continuous walls (hedgerows) about 3 m in height and different width for different harvesting machinery: up to 1.5 m for straddle harvesters; from 1.6 to 3 m for shakers equipped with side-by-side interceptor frame. Trees at 4 × 5 m were trained to small globe vase (GVx4) and kept at 3 m in height. Trees at 5 × 5 m were trained to poly-conic vase (PVx5) and kept at 3.5 m in height. For a detailed description of training forms see Massenti et al., 2022a.

Two self-compensating in-line drippers per plant, delivering 16 L/h, were used for weekly irrigation during the main summer dry period. Trees were deficit irrigated using a water potential threshold of –2.0 MPa (Marino et al., 2018). The total seasonal (4^th^ week of June to 1^st^ week of October) application rate ranged from 500 to 1300 m^3^ ha^-1^ year^-1^, depending on planting density and season. All other conventional cultural cares were the same for all trees in trial. Trees were fertilized with 200 kg/ha of 20-5-10 NPK complex to the soil and with 60 kg of 20-0-20 in the irrigation water, for a total of 130, 25, and 80 g/tree of N, P, and K, respectively. Soil was cultivated at a 10–20 cm depth three times per year in mid-March, at the end of May, and at the end of August.

### 2.2 Experiment layout and measurements

Nine trees for each cultivar (main plot) and planting system (subplot) combination in a split-plot design were selected and properly labeled. Fruit yield (kg/tree), trunk cross-section area (TCSA, cm^-2^), yield efficiency (kg cm^-2^), and yield (t ha^-1^) were determined for each tree as a biological replicate in each of the three years of trial. In 2021 and 2023, canopy width, thickness, and height were recorded at three positions (low, mid, and top) and used to estimate canopy surface and volume and calculate their ratio. Vegetative growth efficiency was calculated as the ratio of canopy volume to TCSA. Amount of pruned wood per tree was also recorded.

Fruit of all nine trees per cultivar and planting system combination were hand harvested at veraison stage and placed in bins for processing within 48 hours. Fruits were weighed and processed with a two-phase mill (Toscana Enologica Mori-TEM) with a working capacity of 400 kg of olives/run specifically built for processing relatively small experimental samples. The oil extracted from each combination of factors was subsequently weighed to determine oil yield and sub-samples taken for chemical analyses. Oil yield efficiencies were calculated on a cm^2^ of TCSA.

### 2.3 Olive oil standard quality

In 2023, the standard oil quality parameters of free acidity (% of oleic acid), peroxide value (mEq O_2_ kg^-1^), extinction coefficients (K232, K270, and ΔK) and fatty acid composition were evaluated in accordance with the regulations of the European Union (Commission Delegated Regulation (EU) 2019/1604).

### 2.4 Phenol compounds

The extraction of phenol compounds in extra virgin olive oil (EVOO) was carried out according to Taticchi et al., 2021. The analysis of phenol compounds was carried out as reported by Selvaggini et al., 2014, with an Agilent Technologies HPLC system model 1100, equipped with a vacuum degasser, a quaternary pump, an autosampler, a thermostated column compartment, a diode array detector (DAD), and a fluorescence detector (FLD) controlled by a ChemStation (Agilent Technologies, Palo Alto, CA, USA) and using a C18 column Spherisorb ODS-1 (250 mm x 4.6 mm, 5 μm particle size) (Waters, Milan, Italy). The quantitative evaluation of the phenols was carried out by means of single calibration curves for each compound, and the results were expressed as mg kg^-1^ of oil.

### 2.5 Volatile compounds

The evaluation of volatile compounds of VOOs was carried out with the headspace–solid phase microextraction (HS-SPME) technique followed by gas chromatography–mass spectrometry (HS-SPME-GC/MS). The sampling of headspace of volatile molecules was done with the SPME fiber (50/30 μm DVB/CAR/PDMS, length 2 cm, StableFlex, Supelco, Inc., Bellefonte, PA, USA). GC/MS analysis was conducted with an Agilent Technologies GC 7890B equipped with a “Multimode Injector” (MMI) 7693A (Agilent Technologies, Santa Clara, CA, USA) and a thermostated PAL3 RSI 120 autosampler equipped with a fiber conditioning module and an agitator (CTC Analytics AG, Zwingen, Switzerland) following the protocol described by Taticchi et al., 2021. Volatile compounds were identified by comparing their mass spectra and retention times with those of authentic reference compounds and with spectra in the NIST 2014 mass spectra library. The quantitation of the volatile molecules was performed using calibration curves for each compound with the internal standard method, and the results were expressed as µg kg^-1^ of oil.

### 2.6 Data analysis

Analysis of variance was used to test for differences among studied factors using Jamovi procedures (version 2.5.4, The Jamovi project, 2023, https://www.jamovi.org). Yield and growth data were analyzed according to a split-plot experimental design, with cultivars as the main plot and planting system as the subplot. Nine trees, as replicates, were selected for each subplot. Ninety-five % confidence intervals were used to show differences among means. Due to the limited quantity of olives and the mill working capacity (400 kg of olives/run), olive oil was extracted in three replicates per cultivar and planting system combination. Data were analyzed by analysis of variance followed by Tukey’s multiple comparison test (P ≤ 0.05).

## 3 Results and discussion

### 3.1 Climate conditions

**Figure 1:**
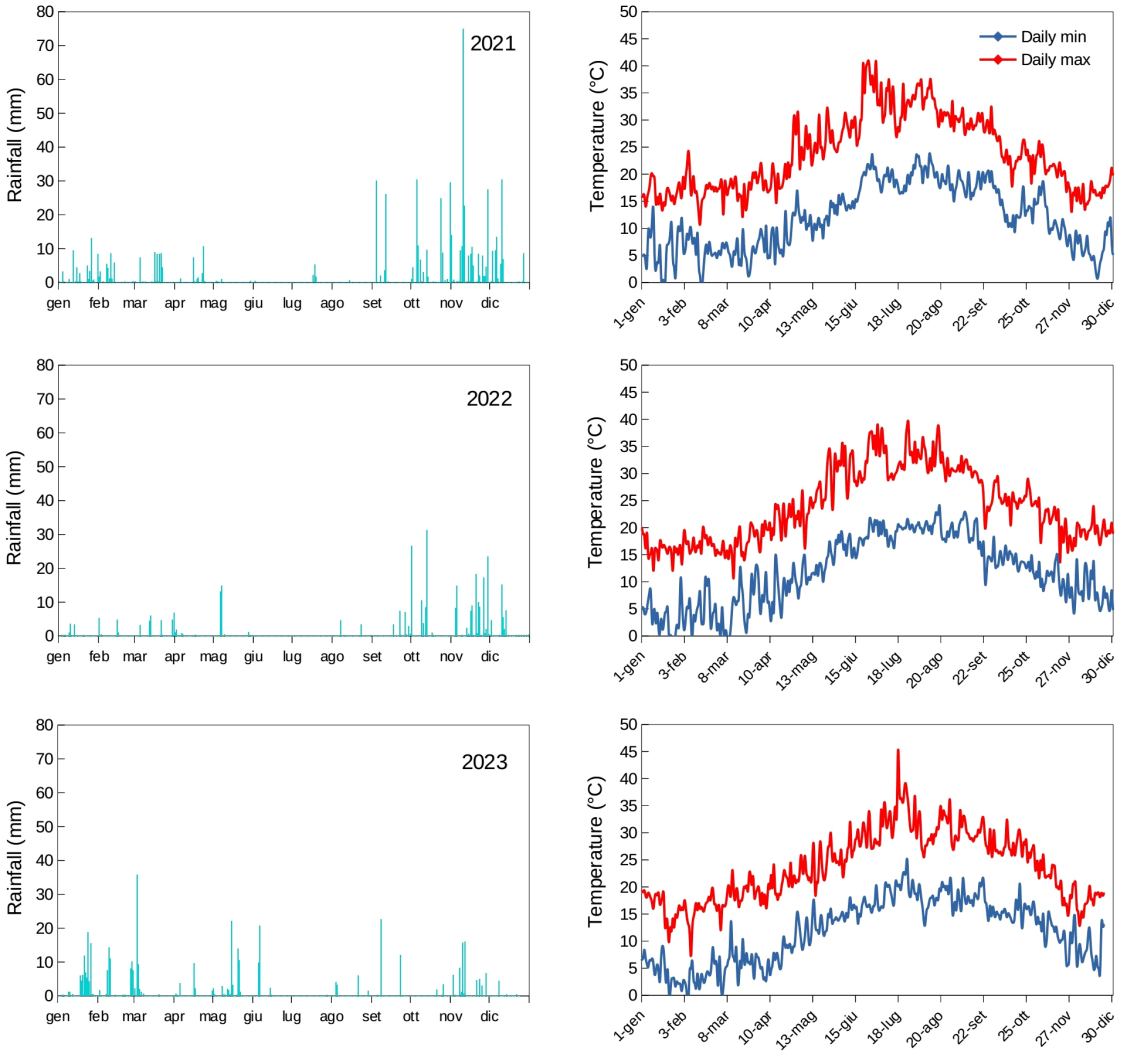
Average daily rainfall (mm) and temperatures (°C) near Sciacca, southwest Sicily (37°31’ N, 13°03’ E, about 120 m a.s.l.) during the three years of trial (2021-2023).

The climate conditions of the area were those typical of the southern Mediterranean regions, with an average annual rainfall of 487 mm for the three years of trial (min 357 mm in 2022, max 668 mm in 2021). The three years of trial were different in terms of total rainfall amount and distribution, and in part also for temperature fluctuations (**Figure 1**). In particular, the year 2023 was the warmest year in fall during harvest (T_max_ 23.1°C) and significantly warmer than the average of the last 30 years (T_max_ 20.1°C). It had higher rainfall and humidity in the mid spring months after the flowering period in early May. The usual trends in the area indicate fall as the main rainy period and this could be observed in 2021 and 2022, while fall 2023 (90 mm) was significantly drier than 2021 (453 mm) and 2022 (254 mm), as well as than the average of the last 30 years (228 mm). Year 2023 can be generally considered as a drastic example of climate change made of several events throughout the year.

### 3.2 Tree growth

**Figure 2:**
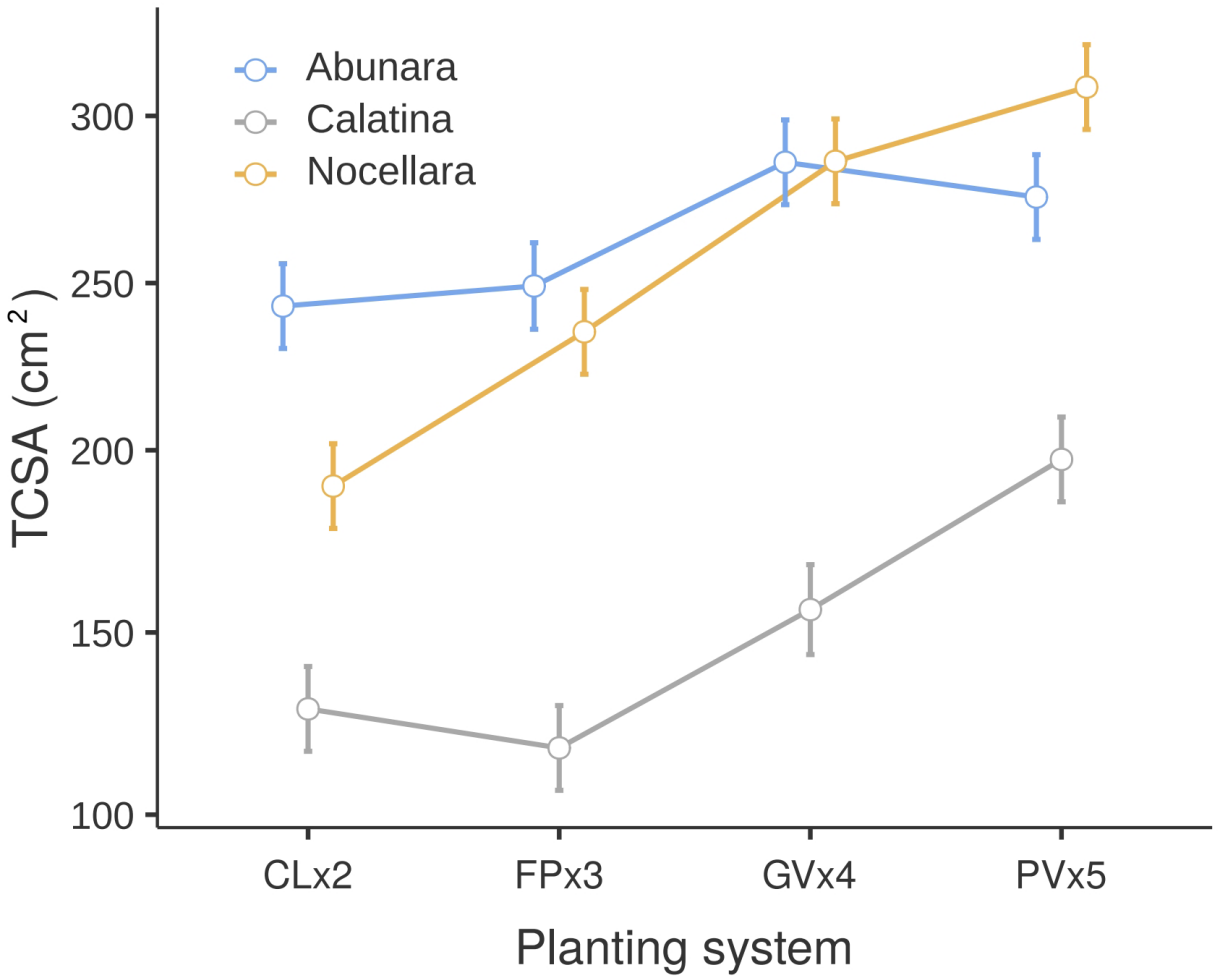
Trunk cross-section area (TCSA, cm^2^) of ‘Abunara’, ‘Calatina’ and ‘Nocellara’ olive trees grown as central leaders at 2 × 5 m (CLx2), free palmette at 3 × 5 m (FPx3), globe vase at 4 × 5 m (GVx4), and polyconic vase at 5 × 5 m (PVx5) near Sciacca, southwest Sicily (37°31’ N, 13°03’ E, about 120 m a.s.l.). Data are averages of the three years of trial (2021-2023). Error bars represent confidence intervals.

As expected, trunk size increased linearly (from 189 to 260 cm^2^) over the three years of trial, regardless of cultivar or growing system. On the average, ‘Abunara’ (270 cm^2^) and ‘Nocellara’ (262 cm^2^) showed significantly greater TCSA than ‘Calatina’ (147 cm^2^), confirming the lowest vigor of the latter already observed in previous trials (Marino et al., 2019; Massenti et al., 2022a). Only ‘Nocellara’ responded linearly to increasing in-row spacing, while ‘Abunara’ and ‘Calatina’ had smaller TCSA in hedgerow systems (CL and FP) than in 3D systems (GV and PV) (**Figure 2**). In a way, this indicates that ‘Abunara’ and ‘Calatina’ respond and adapt better to the combinations of in-row distances and training forms than ‘Nocellara’, which instead suffers tight spacings. Indeed, ‘Nocellara’ trees have the tendency to grow open with wide crotch angles (Marino et al., 2019).

**Figure 3:**
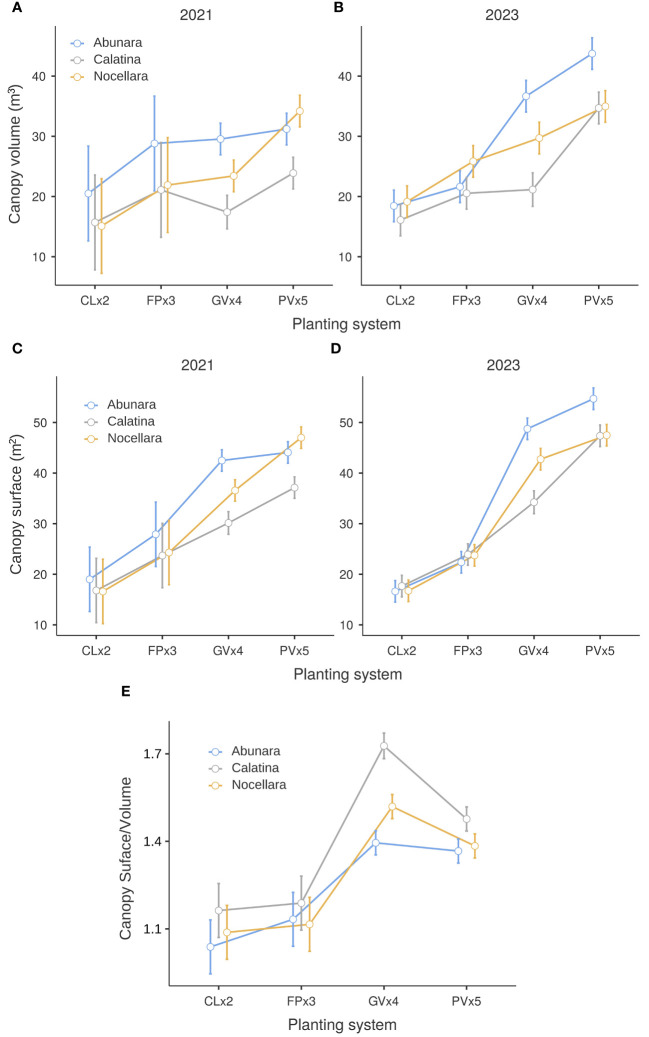
Canopy volume **(A, B)** and surface **(C, D)** in 2021 and 2023 and average of surface/volume ratio **(E)** for 2021 and 2023 of ‘Abunara’, ‘Calatina’ and ‘Nocellara’ olive trees grown as central leaders at 2 × 5 m (CLx2), free palmette at 3 × 5 m (FPx3), globe vase at 4 × 5 m (GVx4), and polyconic vase at 5 × 5 m (PVx5) near Sciacca, southwest Sicily (37°31’ N, 13°03’ E, about 120 m a.s.l.). Error bars represent confidence intervals.

Canopy size measurements were not taken in 2022, and statistical analysis of 2021 and 2023 data revealed significant 2-way interactions (cv x growing system) for all measured variables. In both years, canopy volume was similar in all three cultivars in the two hedgerow systems, CLx2 and FPx3, and this can be explained by the fact that by the 7^th^ year all trees had saturated their allotted space in the hedgerow system. On the other hand, ‘Calatina’ tended to have smaller canopies than the other two cultivars in the growing systems with 3D forms, GVx4 and PVx5, especially in 2021 (**Figures 3A, B**), while ‘Abunara’ tended to have the largest canopies, especially in 2023. As expected, 3D growing systems, and in particular PVx5, tended to have larger canopies than hedgerow systems. On a single tree basis, this is easily explained by larger spaces between trees and better light interception. The exact same trends were observed for canopy surface (**Figures 3C, D**), and generally agree with results obtained by Marino et al., 2019. These results are also in line with what observed in the first years of orchard life (Massenti et al., 2022a) with the exception for more vigorous cultivars that now tend to saturate the allotted space even at 3D systems (lower planting densities). The canopy surface-to-volume ratio (S/V; **Figure 3E**) was higher than 1 in all cultivars and growing systems, indicating a satisfactory level of exposed leaf area for a positive net photosynthetic balance. In trees grown as CLx2, FPx3, and PVx5, S/V was the same for all cultivars, while in trees grown as GVx4, it was highest in ‘Calatina’ and lowest in ‘Abunara’. Those differences observed in the globe vase form were already present in the first years of orchard life (Massenti et al., 2022a) and are the result of a combination of tree vigor (lowest in ‘Calatina’) and growth habit (mainly branching angle, position and bending tendency), suggesting a better use of the occupied space in terms of light interception for ‘Calatina’ under this growing system.

**Figure 4:**
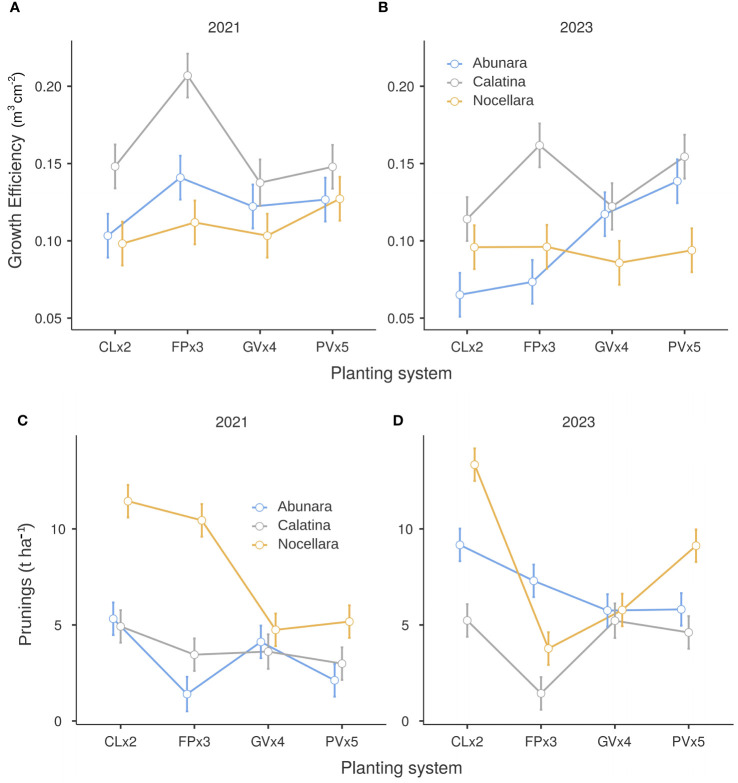
Growth efficiency **(A, B)** and prunings **(C, D)** of ‘Abunara’, ‘Calatina’ and ‘Nocellara’ olive trees grown as central leaders at 2 × 5 m (CLx2), free palmette at 3 × 5 m (FPx3), globe vase at 4 × 5 m (GVx4), and polyconic vase at 5 × 5 m (PVx5) near Sciacca, southwest Sicily (37°31’ N, 13°03’ E, about 120 m a.s.l.) in 2021 and 2023. Error bars represent confidence intervals.

Growth efficiency in terms of m^3^ of canopy per cm^2^ of TCSA was definitely in favor of ‘Calatina’, especially in the higher density hedgerow systems in both years (**Figures 4A, B**). No difference among cultivars was found in the lower density 3D systems. The results from the hedgerow systems agree with those observed at the young orchard stages (Massenti et al., 2022a), while in mature 3D systems, ‘Abunara’ started to perform well like ‘Calatina’ as the above ground structures started to fill the allotted spaces. ‘Nocellara’ was the least growth efficient in 3D growing systems. As for the amount of pruning wood per hectare, in 2021, ‘Nocellara’ showed the greatest amounts in the hedgerow systems, indicating poor adaptability to these systems (**Figure 4C**). In 2023, ‘Calatina’ showed the least amount of prunings in the hedgerow systems, while ‘Nocellara’ the greatest amounts in CLx2 and PVx5 (**Figure 4D**). Overall, ‘Calatina’ exhibited a better growth performance and less need for pruning than ‘Nocellara’ in the higher density hedgerow systems confirming the trends observed during the early stages of orchard life. ‘Abunara’, despite its high vigor, performed better that ‘Nocellara’ in 2021 and in 2023 only in 3D growing systems. This may be due to the more upright growth habit and less dense canopies of ‘Abunara’ compared to ‘Nocellara’ (Caruso et al., 2007).

### 3.3 Fruit yield

**Figure 5:**
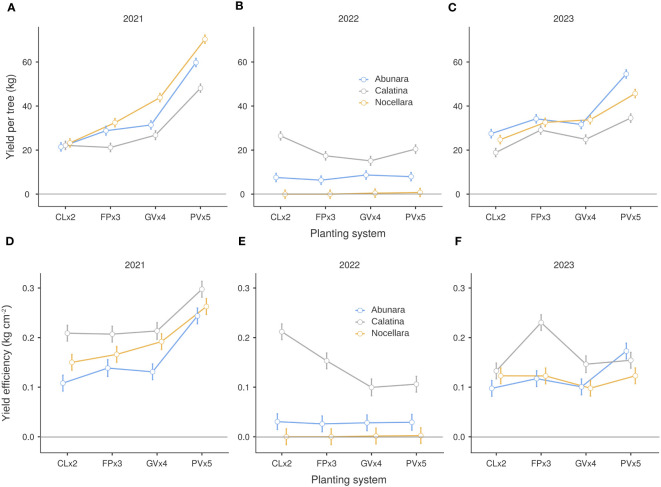
Yield per tree **(A–C)** and yield efficiency **(D–F)** of ‘Abunara’, ‘Calatina’ and ‘Nocellara’ olive trees grown as central leaders at 2 × 5 m (CLx2), free palmette at 3 × 5 m (FPx3), globe vase at 4 × 5 m (GVx4), and polyconic vase at 5 × 5 m (PVx5) near Sciacca, southwest Sicily (37°31’ N, 13°03’ E, about 120 m a.s.l.) in 2021, 2022 and 2023. Error bars represent confidence intervals.

In 2021, fruit yield per tree of ‘Nocellara’ increased linearly with in-row distance and was significantly higher than yield of ‘Calatina’ in FPx3, GVx4, and PVx5 (**Figure 5A**). Yields of ‘Abunara’ were in the middle between ‘Nocellara’ and ‘Calatina’. Yields of ‘Abunara’ and ‘Calatina’ were similar in CLx2, FPx3, and GVx4 and highest in PVx5. In 2022, trees of ‘Nocellara’, and in part of ‘Abunara’, were pruned heavily to fit back into the allotted spaces of each growing system. In particular, heavy pruning of those cultivars was necessary in the hedgerow systems, as branches were growing fast toward the inter-row and relatively large limbs could interfere with straddle harvesters or even with side-by-side intercepting frames of trunk shakers. As a result of the heavy pruning, but also for a greater alternate bearing tendency of the more vigorous cultivars (Massenti et al., 2022a), fruit yields of ‘Nocellara’ and ‘Abunara’ trees were generally low in 2022, and nearly canceled in ‘Nocellara’ (**Figure 5B**). In this year, fruit yield per tree was highest in ‘Calatina’, intermediate in ‘Abunara’, and lowest in ‘Nocellara’, with nearly no effect of growing system. In 2023, trees at PVx5 gave again the highest yields per tree as expected. Differences among cultivars could be appreciated only in the two 3D growing systems where ‘Calatina’ showed the lowest yields per tree (**Figure 5C**). As already observed during the early orchard life, the two 3D growing systems showed a higher degree of alternate bearing compared to hedgerow systems (Massenti et al., 2022a).

‘Calatina’ was the most yield efficient in several cases, especially in the two hedgerow systems, and in all three years when grown at FPx3 (**Figures 5D–F**). ‘Abunara’ and ‘Nocellara’ were very often equally efficient. In 2021 and 2023, ‘Abunara’ was more efficient at PVx5 than in the other growing systems (**Figures 5D, F**). These results in part confirm the observations done during the early orchard life and in part show that at orchard maturity, also more vigorous cultivars grown as 3D systems start to perform well becoming as or even more yield efficient than hedgerow systems.

**Figure 6:**
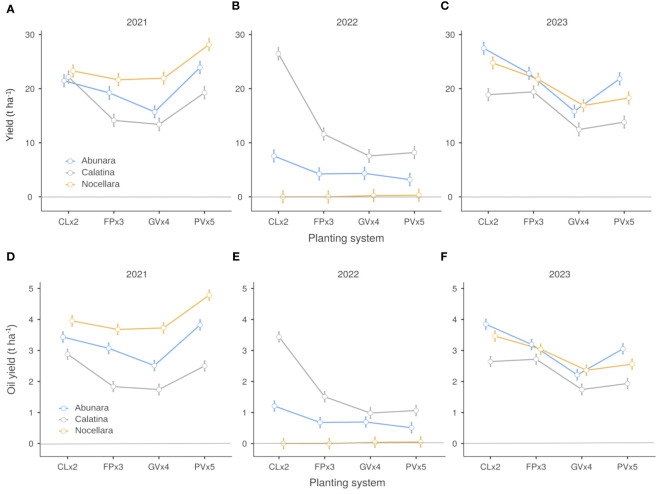
Fruit yield **(A–C)** and oil yield **(D–F)** per hectare of ‘Abunara’, ‘Calatina’ and ‘Nocellara’ olive trees grown as central leaders at 2 × 5 m (CLx2), free palmette at 3 × 5 m (FPx3), globe vase at 4 × 5 m (GVx4), and polyconic vase at 5 × 5 m (PVx5) near Sciacca, southwest Sicily (37°31’ N, 13°03’ E, about 120 m a.s.l.) in 2021, 2022 and 2023. Error bars represent confidence intervals.

When fruit yields were calculated on a hectare basis, the higher density hedgerow growing systems tended to perform at the same level (2021, **Figure 6A**) or even better than the lower density 3D systems (2023, **Figure 6C**). In 2022, the effect of the growing system was evident only in ‘Calatina’, where higher density hedgerows (especially CLx2) produced 2-3 times more than 3D growing systems (**Figure 6B**). The same exact trends were observed for oil yield per hectare (**Figures 6D–F**), with only more marked differences among cultivars in 2021 (**Figure 6D**). With the exception of 2022 when trees were heavily pruned, yields of all trial combinations were generally satisfactory (12-28 t ha^-1^ of olives and 1.5-5 t ha^-1^ of olive oil), indicating that, with the due differences, all four growing systems were adequate for sustainable olive growing of the selected cultivars in the region. In the early stages of orchard life, ‘Calatina’ had outperformed in terms of yield per ha both ‘Abunara’ and ‘Nocellara’ in all growing systems except PVx5 (Massenti et al., 2022a). At the mature stage, we can clearly see that all three cultivars can produce well, each one in a specific combination of training form and planting layout, i.e. under the most appropriate growing system. For instance, we can say that ‘Calatina’ performs best under higher density hedgerow systems, while ‘Nocellara’ performs best under lower density 3D systems like PVx5. ‘Abunara’ seems to perform well both under CLx2 and PVx5, indicating a good level of plasticity most likely due to a more upright growth habit and a less dense canopy (Caruso et al., 2007).

**Figure 7:**
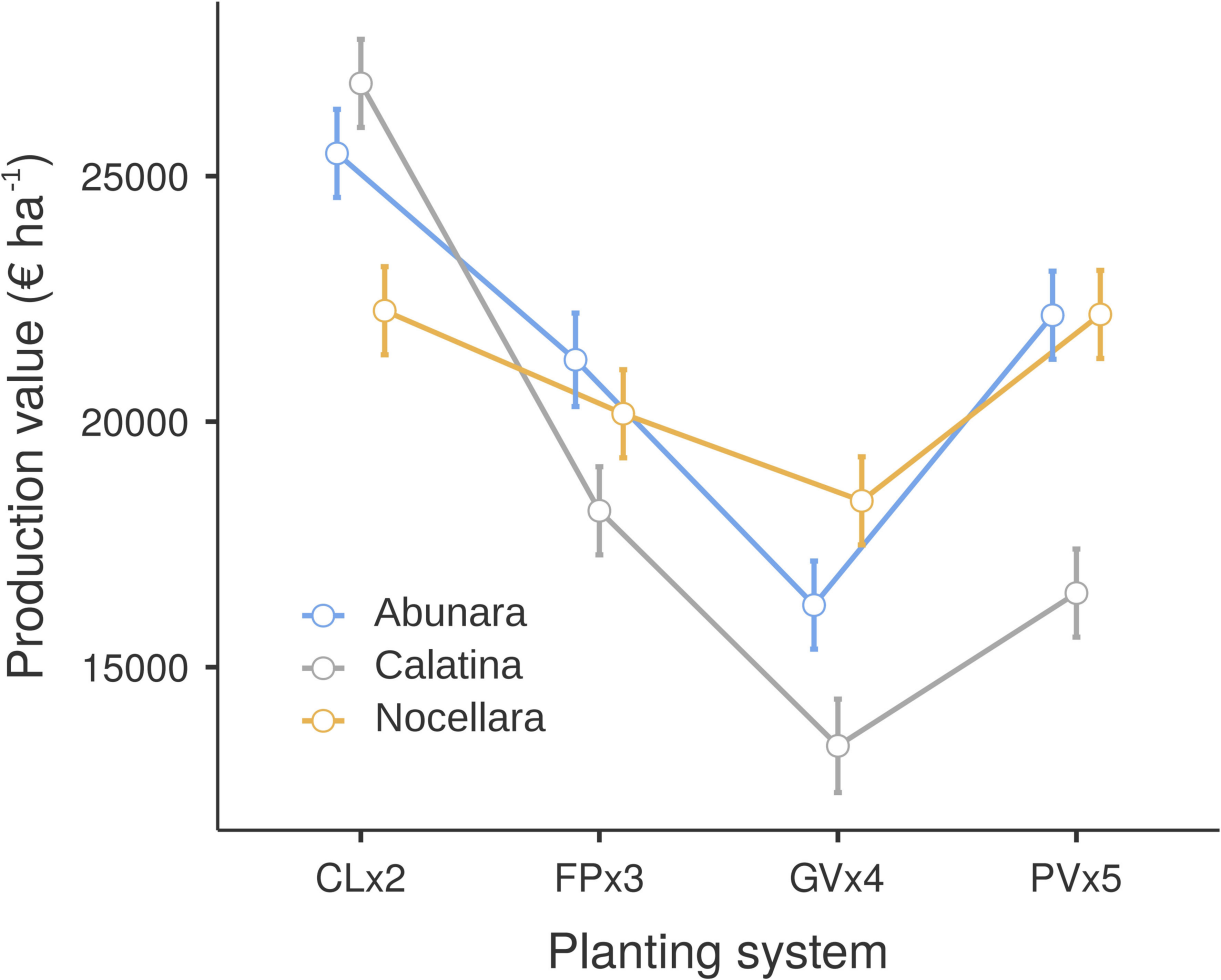
Olive oil production value per hectare of ‘Abunara’, ‘Calatina’ and ‘Nocellara’ olive trees grown as central leaders at 2 × 5 m (CLx2), free palmette at 3 × 5 m (FPx3), globe vase at 4 × 5 m (GVx4), and polyconic vase at 5 × 5 m (PVx5) near Sciacca, southwest Sicily (37°31’ N, 13°03’ E, about 120 m a.s.l.). Average oil yield of 2021, 2022 and 2023 and oil price at 9 € kg^-1^. Error bars represent confidence intervals.

Considering the average yield of the three years of trial and an average price of the olive oil in the area of about 9 euro per kg, ‘Abunara’ and ‘Calatina’ gave the highest production values when grown to CLx2 (25,463 and 26,889 € ha^-1^, respectively) and the lowest production value when grown to GVx4 (16,264 and 13,397 € ha^-1^, respectively) (**Figure 7**). In ‘Nocellara’, the differences in production value among growing systems were much less evident, with CLx2 and PVx5 (22,259 and 22,183 € ha^-1^, respectively) giving higher values than GVx4 (18,387 € ha^-1^).

### 3.4 Oil quality

In 2023, the Jaen index suggested that olives of ‘Calatina’ were generally riper at harvest than those of ‘Abunara’ and ‘Nocellara’ (2.9, 0.83 and 0.69, respectively; P < 0.001). Also, CLx2 tended to induce earlier fruit maturation, followed by PVx5, GVx4, and FPx3 according to the Jaen indexes (1.83, 1.48, 1.24, and 1.33, respectively), although differences among planting systems were not statistically significant (P = 0.943). Nevertheless, detailed observations, especially of ‘Calatina’ olives or for Jaen indexes below 4, indicated that there was really poor correspondence between skin and flesh color and real ripening degree of the fruit. ‘Calatina’ fruit was indeed getting purple/black color on the skin relatively early compared to other cultivars, but it was not showing any sign of ripening (color or firmness) in the pulp or loss of peduncle detachment force (data not shown). This ultimately suggested that Jaen index needs to be monitored in a more precise way, perhaps using image analysis or other appropriate technologies, and possibly calibrated for each cultivar; in alternative, it could only be useful for comparing different timing or treatments within the same cultivar. In addition, ‘Abunara’ olives tended to have a low peduncle detachment force and drop easily during maturation, so they needed to be harvested sooner than ‘Calatina’ olives, which on the contrary could stay longer on the tree due to a high peduncle detachment force (data not shown).

All the oils obtained in 2023 from the three cultivars and four growing systems can be considered high-quality extra-virgin olive oils as they showed values well within the legal limits fixed for the extra virgin olive oil (**Table 1**). Oils from ‘Abunara’ and ‘Nocellara’ had a generally lower percentage of acidity than oils from ‘Calatina’, and this may be related to the higher degree of maturation of ‘Calatina’ olives suggested by the Jaen index. In ‘Abunara’, oils from trees grown as CLx2 and GVx4 had lower acidity than oils from trees grown as FPx3 and PVx5. In ‘Calatina’, oils from the trees grown as GVx4 had the lowest acidity, followed in the order by oils from trees grown as FPx3, PVx5, and CLx2. Higher values of ΔK were found in ‘Abunara’ grown as CLx2 and ‘Nocellara’ grown as GVx4. Overall, the observed differences may be considered relatively minor differences as all oils exhibited values well within the legal limits fixed for the extra virgin olive oil and particularly for the PGI Sicily olive oil. Indeed, no major difference was observed in oils from the same trees even during the early stages of orchard life (Massenti et al., 2022a).

**Table 1:** Standard quality parameters of virgin olive oils from ‘Abunara’, ‘Calatina’ and ‘Nocellara’ olive trees grown as central leaders at 2 × 5 m (CLx2), free palmette at 3 × 5 m (FPx3), globe vase at 4 × 5 m (GVx4), and polyconic vase at 5 × 5 m (PVx5) near Sciacca, southwest Sicily (37°31’ N, 13°03’ E, about 120 m a.s.l.).

		Acidity (%)	Peroxide Value (meq O_2_ kg^-1^ Oil)	K_232_	K_270_	ΔK
Abunara	CLx2	0.21 b	8.9	1.64	0.080	-0.004 a
	FPx3	0.27 a	9.8	1.76	0.092	-0.002 b
	GVx4	0.22 b	9.6	1.74	0.083	-0.002 b
	PVx5	0.28 a	8.8	1.60	0.090	-0.002 b
Calatina	CLx2	0.56 a	8.9	1.43	0.105	-0.002
	FPx3	0.39 c	7.3	1.60	0.111	-0.002
	GVx4	0.28 d	8.5	1.67	0.114	-0.002
	PVx5	0.47 b	8.0	1.56	0.104	-0.003
Nocellara	CLx2	0.23	7.6	1.62	0.086	-0.003 ab
	FPx3	0.24	7.4	1.59	0.084	-0.002 b
	GVx4	0.25	7.2	1.68	0.092	-0.004 a
	PVx5	0.24	7.3	1.65	0.089	-0.002 b

Data are expressed as means (oils from the 2023 harvest). When present, different letters indicate significant differences among growing systems and within each cultivar (Tukey’s test at P < 0.05).

The growing system did not have any effect on fatty acid composition, while there were significant differences among cultivars; therefore, data from different growing systems were pooled together for each cultivar. ‘Calatina’ exhibited the lowest contents in palmitic and palmitoleic acids and the highest contents in oleic, linolenic and eicosenoic acids (**Table 2**). Overall, ‘Calatina’ had the lowest content in saturated fatty acids and the highest in mono-unsaturated fatty acids, indicating the production of oils with high health values and oxidative stability (Piroddi et al., 2017). On the other hand, ‘Abunara’ had the highest content in poly-unsaturated fatty acids and ‘Nocellara’ the highest content in saturated fatty acids. These results confirm the high suitability of ‘Calatina’ trees for making high-quality olive oils in dry and warm areas like the ones described in the present work, in agreement with results obtained during the early stages of orchard life (Massenti et al., 2022a).

**Table 2:** Fatty acid composition (%) of virgin olive oils from ‘Abunara’, ‘Calatina’ and ‘Nocellara’ olive trees grown near Sciacca, southwest Sicily (37°31’ N, 13°03’ E, about 120* m* a.s.l.).

	Abunara	Calatina	Nocellara
Myristic (C14:0)	n.d.	n.d.	n.d.
Palmitic (C16:0)	12.1 b	11.4 c	13.4 a
Palmitoleic (C16:1)	1.14 a	0.83 c	1.01 b
Margaric (C17:0)	0.02 c	0.06 a	0.03 b
Margaroleic (C17:1)	0.03 b	0.07 a	0.05 b
Stearic (C18:0)	2.85 c	3.09 b	3.53 a
Oleic (C18:1 ω-9)	69.1 c	72.9 a	72.3 b
Linoleic (C18:2 ω-6)	13.2 a	10.1 b	8.12 c
Linolenic (C18:3 ω-3)	0.79 b	0.84 a	0.79 b
Arachidic (C20:0)	0.42 b	0.41 b	0.44 a
Eicosenoic (C20:1)	0.29 b	0.33 a	0.27 b
Behenic (C22:0)	n.d.	n.d.	n.d.
Lignoceric (C24:0)	n.d.	0.08	0.08
SFA	15.4 b	15.0 c	17.4 a
MUFA	70.6 c	74.1 a	73.7 b
PUFA	14.0 a	10.9 b	8.90 c

Data are expressed as means (oils from the 2023 harvest). Different letters indicate significant differences among cultivars for each compound (Tukey’s test at P < 0.05).

n.d., not dectected.

Total phenol content was generally low in all cultivars and did not agree with what observed in previous studies in terms of differences among the three cultivars (Marino et al., 2017, 2019; Massenti et al., 2022a). This may be attributed to the relatively high temperatures and dry conditions of fall 2023 (**Figure 1**) as well as to differences in crop load. The present findings confirm the strong effect of climate factors, which are able to modify the phenol profiles and total contents from one season to another as already observed in the same trees at the early stages of orchard life (Massenti et al., 2022a). Differences among growing systems were found for many bioactive compounds, but they were cultivar dependent (**Table 3**). The most represented phenols were the oleuropein derivatives, especially oleacein; less represented, but still with significant differences among growing systems, were the ligstroside derivatives, such as oleocanthal; no effect of growing system was observed on lignans. In particular, in ‘Abunara’, the highest amount of oleuropein derivatives was found in CLx2, whereas in ‘Calatina’ and ‘Nocellara’ the highest amount of oleuropein derivatives was found in FPx3. Regardless of cultivar, tyrosol and hydroxytyrosol content was higher in the CLx2 planting system. This was likely a symptom of stress related to increased competition for water and nutrients at closer tree spacings. In these cases, plants may activate defense and adaptation mechanisms to improve their survival, which may include the production of phenolic compounds. In terms of total phenols, ‘Abunara’ oils exhibited again the highest amount in CLx2, while ‘Calatina’ and ‘Nocellara’ oils exhibited the highest amount in FPx3 and PVx5. Higher total phenols were also found at higher tree densities in a study conducted on ‘Cerasuola’ and ‘Koroneiki’ olive (Grilo et al., 2021). Unexpectedly, the observed differences did not seem to be related to the degree of maturation of the harvested olives measured by the Jaen index. Yet, as mentioned before, Jaen index values below 4 are relatively poor indicators of olive ripeness. In addition or in alternative, other factors like canopy shape/size and fruit position in the canopy (light interception), inner micro-environment due to canopy density (temperatures within the canopy), and crop load may have a role in determining the total abundance of bioactive compounds in the oils (Grilo et al., 2019, 2021). Indeed, a negative relationship is expected between total bioactive compounds and tree crop load and trees grown as CLx2 were those with the lowest yield per tree.

**Table 3:** Phenolic composition (mg kg^−1^) of virgin olive oils from ‘Abunara’, ‘Calatina’ and ‘Nocellara’ olive trees grown as central leaders at 2 × 5* m* (CLx2), free palmette at 3 × 5* m* (FPx3), globe vase at 4 × 5* m* (GVx4), and polyconic vase at 5 × 5* m* (PVx5) near Sciacca, southwest Sicily (37°31’ N, 13°03’ E, about 120* m* a.s.l.).

	Abunara			
	CLx2	FPx3	GVx4	PVx5
Hydroxytyrosol (3,4-DHPEA)	4.6 a	0.9 c	3 b	0.8 c
Tyrosol (p-HPEA)	4.6 a	1.0 c	1.7 b	1.2 bc
Vanillic acid	0.1	0.1	0.1	0.1
Oleacein (3,4-DHPEA-EDA)	197 a	133 b	115 c	120 bc
Oleocanthal (p-HPEA-EDA)	28.4 a	22.2 b	21 b	22.8 b
(+)-1-Acetoxypinoresinol	15.5	15.4	15.3	15.8
(+)-Pinoresinol	6.1	6.5	6	6.6
Oleuropein aglycone (3,4-DHPEA-EA)	24.7	24	23.5	24.5
ligstroside aglycone	4.4	4.7	4.2	3.9
Total phenols	285 a	208 b	190 c	196 bc
Sum of oleuropein derivatives	226 a	158 b	142 c	145 bc
Sum of ligstroside derivatives	37.4 a	27.9 b	26.9 b	27.9 b
Sum of lignans	21.5	21.9	21.3	22.4
	Calatina			
	CLx2	FPx3	GVx4	PVx5
Hydroxytyrosol (3,4-DHPEA)	46.8 a	21.5 b	5.1 d	17.7 c
Tyrosol (p-HPEA)	15.8 a	6.4 b	2.0 c	6.0 b
Vanillic acid	0.3	0.3	0.3	0.3
Oleacein (3,4-DHPEA-EDA)	122 b	175 a	168 a	170 a
Oleocanthal (p-HPEA-EDA)	30.5 a	28.7 ab	25.3 b	28.4 ab
(+)-1-Acetoxypinoresinol	11.6	11.0	11.0	10.8
(+)-Pinoresinol	7.6	8.1	7.9	8.0
Oleuropein aglycone (3,4-DHPEA-EA)	10.5 b	13.7 b	25.4 a	13.9 b
Ligstroside aglycone	9.2	9.5	8.7	8.9
Total phenols	255 b	274 a	253 b	264 ab
Sum of oleuropein derivatives	180 c	210 a	198 b	202 b
Sum of ligstroside derivatives	55.5 a	44.6 b	36.0 c	43.3 b
Sum of lignans	19.2	19.1	18.9	18.8
	Nocellara			
	CLx2	FPx3	GVx4	PVx5
Hydroxytyrosol (3,4-DHPEA)	23.6 a	7.4 c	5.3 d	9.3 b
Tyrosol (p-HPEA)	7.3 a	2.5 b	2.2 b	3.3 b
Vanillic acid	0.29 a	0.30 a	0.26 b	0.30 a
Oleacein (3,4-DHPEA-EDA)	190 b	215 a	194 b	200 b
Oleocanthal (p-HPEA-EDA)	29.1 b	36.4 a	32.1 b	37.8 a
(+)-1-Acetoxypinoresinol	25.2	24.9	23.8	24.5
(+)-Pinoresinol	6.8	7.4	6.3	7.0
Oleuropein aglycone (3,4-DHPEA-EA)	20.1	22.3	26.6	22.9
Ligstroside aglycone	5.5 b	6.2 a	5.1 b	6.6 a
Total phenols	308 ab	322 a	296 b	312 a
Sum of oleuropein derivatives	234 b	245 a	226 c	233 b
Sum of ligstroside derivatives	41.9	45.1	39.4	47.7
Sum of lignans	31.9	32.3	30.1	31.5

Data are expressed as means (oils from the 2023 harvest). Different letters indicate significant differences among growing systems and within each cultivar (Tukey’s test at P < 0.05).

Regarding the volatile fraction, oils from ‘Nocellara’ showed the highest contents of aldehydes (7738 µg kg^−1^) and alcohols (7080 µg kg^−1^), and the lowest contents of esters (116 µg kg^−1^); no differences among cultivars were observed for ketons. Again, the effect of the growing system was cultivar dependent (**Table 4**). Both, trans-2-hexenal, responsible for the “cut grass” sensory note, and hexenyl acetate, associated with the “floral” sensory note, tended to be lowest in oils from trees grown at CLx2, which had also the most mature olives at harvest. On the contrary, the overall aromatic composition of oils from GVx4 indicates that drupes obtained with this growing system are characterized by high levels of lipoxygenase activity, leading to the production of olive oils with a high level of positive sensory notes such as cut grass and floral. In this case, tree crop load alone does not seem to explain the differences in volatile composition or abundance and probably a combination of light interception and degree of maturation may play a major role.

**Table 4:** Volatile composition (µg kg^−1^) of virgin olive oils from ‘Abunara’, ‘Calatina’ and ‘Nocellara’ olive trees grown as central leaders at 2 × 5* m* (CLx2), free palmette at 3 × 5* m* (FPx3), globe vase at 4 × 5* m* (GVx4), and polyconic vase at 5 × 5* m* (PVx5) near Sciacca, southwest Sicily (37°31’ N, 13°03’ E, about 120* m* a.s.l.).

	Abunara				Calatina				Nocellara			
	CLx2	FPx3	GVx4	PVx5	CLx2	FPx3	GVx4	PVx5	CLx2	FPx3	GVx4	PVx5
	*Aldehydes*											
Pentanal	58 c	93 a	75 b	56 c	54 ab	66 a	51 b	57 ab	64 a	58 a	44 b	60 a
(*E*)-2-Pentenal	30 b	39 a	29 b	42 a	10 d	16 c	40 a	26 b	7 b	14 ab	20 ab	27 a
Hexanal	672 c	1028 b	1014 b	1168 a	377 b	509 a	499 a	486 a	1306 c	1659 ab	1759 a	1608 b
(*E*)-2-Hexenal	3554	3990	3695	3744	3234 c	3631 bc	4289 a	3952 ab	3111 b	7412 a	5713 a	6889 a
(*Z*)-3-Hexenal	111 b	175 a	151 ab	150 ab	10 c	87 ab	104 a	69 b	163 b	333 a	275 a	323 a
(*E,E*)-2,4-Hexadienal	14 b	20 a	17 ab	17 ab	4 d	15 b	27 a	11 c	18 c	33 a	27 b	28 ab
Sum of C_5_ and at C_6_	4439 b	5345 a	4981 a	5177 a	3689 c	4324 b	5010 a	4601 ab	4669 c	9509 a	7838 b	8935 a
	*Alcohols*											
1-Pentanol	52 a	14 b	54 a	14 b	82 a	82 a	32 b	50 b	149 a	43 b	52 b	56 b
1-Penten-3-ol	577 a	542 ab	528 b	558 ab	392 c	577 a	592 a	430 b	685 a	600 c	647 b	599 c
(*E*)-2-Penten-1-ol	40 a	31 c	35 b	33 bc	46	44	42	45	62 a	42 c	49 b	41 c
(*Z*)-2-Penten-1-ol	273 a	248 b	243 b	256 ab	203 c	333 a	352 a	248 b	390 a	333 c	367 b	331 c
1-Hexanol	435 b	117 c	685 a	143 c	586 b	1142 a	257 d	425 c	1184 a	296 c	490 b	449 b
(*E*)-2-Hexen-1-ol	405 a	228 b	236 b	226 b	2111 a	1721 b	900 c	2203 a	1798 a	454 c	754 b	505 c
(*Z*)-3-Hexen-1-ol	1604 b	943 c	1872 a	941 c	1557 d	2926 a	1919 c	2148 b	8426 a	2509 d	3778 b	3230 c
Sum of C_5_ and at C_6_	3386 a	2123 c	3653 b	2171 c	4977 c	6825 a	4094 d	5549 b	12694 a	4277 d	6137 b	5211 c
	*Esters*											
Ethyl acetate	18 a	10 c	12 b	12 b	–	71 b	10 c	88 a	57 a	13 d	42 b	16 c
Hexyl acetate	58 b	57 b	112 a	65 b	53 c	81 b	76 b	97 a	37	32	30	34
(*Z*)-3-Hexenyl acetate	433 b	249 c	460 a	202 d	151 c	450 b	560 a	463 b	79 b	89 a	74 b	88 a
Sum of esters at C_6_	491 b	306 c	572 a	267 d	204 c	531 b	636 a	560 b	116 ab	121 a	104 b	122 a
	*Ketones*											
3-Pentanone	300	291	314	285	327 b	563 a	362 b	564 a	260 b	306 ab	344 a	393 a
1-Penten-3-one	489	518	529	530	367 b	205 c	558 a	230 c	304 b	428 a	379 a	375 a
6-Methyl-5-hepten-2-one	n.d.	n.d.	n.d.	n.d.	n.d.	n.d.	n.d.	n.d.	n.d	n.d	n.d	n.d
Sum of C_5_ and at C_8_	789	809	843	815	694 c	768 bc	920 a	794 b	564 b	734 a	723 a	768 a

Data are expressed as means (oils from the 2023 harvest). Different letters indicate significant differences among growing systems and within each cultivar (Tukey’s test at P < 0.05).

## 4 Conclusions

As expected, ‘Abunara’ and ‘Nocellara’ shared some common characteristics in terms of TCSA, yield per tree, yield efficiency, and canopy size, probably due to similar vigor and higher than ‘Calatina’. The latter presented some interesting characteristics related to production efficiency, such as growth and yield efficiency as well as fruit production value, especially when trained to hedgerows. Low vigor and small canopy size are other important characteristics of ‘Calatina’ because they allow less need of pruning and easier mechanization of harvesting and pruning, ensuring considerable economic savings. ‘Calatina’ oils also presented very interesting quality traits, especially when grown at FPx3. This, presumably, is thanks to the low vigor of the cultivar and the shape of the canopies, suggesting in turn a better light interception linked with positive outcomes on the product quantity and quality. A better production in terms of higher content of un-saturated fatty acids, phenols, and volatile compounds is very important because nowadays consumers’ preference is strongly directed toward healthier products. In addition, the uniqueness of the oils produced with these combinations of cultivars and growing systems, expressed by their phenol and aromatic profiles, guarantees their access to specialty markets with good prices and profits for the producers.

Along with the results obtained during the early stages of orchard life, the present study with mature orchards highlights two major outcomes: 1) proper combinations of different cultivars (vigor, growth habit), planting densities and training forms may all result in efficient intensive pedestrian systems for growing olive in areas where SHD systems cannot be profitable due to agronomic and environmental limitations (water shortage, steep sloping sites, small farm size, etc.); 2) intensive pedestrian growing systems can exploit olive biodiversity by allowing the use of available local genotypes (even minor or neglected cultivars) and may represent an effective and sustainable solution against unexpected climate changes and associated emerging diseases. The above is equal to say that intensive pedestrian olive growing systems may represent a very flexible alternative to SHD systems: not one for all, but one for each! For example, higher vigor cultivars and lower density 3D systems harvested with trunk shakers can be used in steep sloping and drier sites, while lower vigor cultivars and higher density hedgerow systems harvested with straddle machines can be used in large flat (or gently sloping) areas with available irrigation water.

## Data availability statement

The original contributions presented in the study are included in the article/supplementary material. Further inquiries can be directed to the corresponding author.

## Author contributions

RM: Funding acquisition, Investigation, Writing – original draft, Writing – review & editing. AI: Data curation, Investigation, Writing – review & editing. AC: Data curation, Formal analysis, Investigation, Writing – review & editing. VI: Data curation, Investigation, Writing – review & editing. RL: Formal analysis, Visualization, Writing – original draft, Writing – review & editing. MS: Resources, Supervision, Validation, Writing – review & editing. RS: Data curation, Formal analysis, Methodology, Writing – review & editing. TC: Conceptualization, Funding acquisition, Methodology, Project administration, Supervision, Writing – review & editing.
